# Microbial Community Analysis in the Roots of Aquatic Plants and Isolation of Novel Microbes Including an Organism of the Candidate Phylum OP10

**DOI:** 10.1264/jsme2.ME11288

**Published:** 2011-12-06

**Authors:** Yasuhiro Tanaka, Hideyuki Tamaki, Hiroaki Matsuzawa, Masahiro Nigaya, Kazuhiro Mori, Yoichi Kamagata

**Affiliations:** 1 Interdisciplinary Graduate School of Medicine and Engineering, University of Yamanashi, 4–3–11 Takeda, Kofu, Yamanashi 400–8511, Japan; 2 Bioproduction Research Institute, National Institute of Advanced Industrial Science and Technology (AIST), Central 6, Higashi 1–1–1, Tsukuba, Ibaraki 305–8566, Japan; 3 Bioproduction Research Institute, National Institute of Advanced Industrial Science and Technology (AIST), 2–17–2–1 Tsukisamu-higashi, Toyohira-ku, Sapporo, Hokkaido 062–8517, Japan; 4 Research Faculty of Agriculture, Hokkaido University, Kita-ku, Sapporo, Hokkaido 060–8589, Japan

**Keywords:** aquatic plant, microbial community, novel microbe, *Armatimonadetes*

## Abstract

A number of molecular ecological studies have revealed complex and unique microbial communities in various terrestrial plant roots; however, little is known about the microbial communities of aquatic plant roots in spite of their potential use for water quality improvement in aquatic environments (*e.g.* floating treatment wetland system). Here, we report the microbial communities inhabiting the roots of emerged plants, reed (*Phragmites australis*) and Japanese loosestrife (*Lythrum anceps*), collected from a floating treatment wetland in a pond by both culture-independent and culture-dependent approaches. Culture-independent analysis based on 16S rRNA gene sequences revealed that the microbial compositions between the two aquatic plant roots were clearly different (*e.g.* the predominant microbe was *Betaproteobacteria* for reed and *Alphaproteobacteria* for Japanese loosestrife). In comparisons of microbial communities between the plant roots and pond water taken from near the plants, the microbial diversity in the plant roots (*e.g.* 4.40–4.26 Shannon-Weiner index) were higher than that of pond water (*e.g.* 3.15 Shannon-Weiner index). Furthermore, the plant roots harbored 2.5–3.5 times more phylogenetically novel clone phylotypes than pond water. The culture-dependent approach also revealed differences in the microbial composition and diversity among the two plant roots and pond water. More importantly, compared to pond water, we succeeded in isolating approximately two times more novel isolate phylotypes, including a bacterium of candidate phylum OP10 (recently named *Armatimonadetes*) from the plant roots. These findings suggest that aquatic plants roots are significant sources for a variety of novel organisms.

Many studies of the microbial communities associated with the roots of terrestrial plants have so far been reported (8, 10, 11, 18, 24, 32, 35, 36, 49), suggesting that the microbes might exert a beneficial, neutral or deleterious influence on plant growth. Some of these studies also revealed that the roots of different plant species interacted with different microbial communities (10, 11, 35, 36, 49), and that the growth and activity of microbial populations in the area, which is adjacent to the root system (rhizosphere) and on the external surface of the roots (rhizoplane) are, in general, greater than that root-free soil due to root exudates such as amino acids, sugars and growth factors (11, 26, 36).

Like terrestrial plants, aquatic plants living in water environments also closely interact with microbial communities on their roots. Some important microbial processes such as plant growth promotion, nitrification, denitrification, and remediation of contaminants are significantly active in aquatic plant roots as observed in terrestrial plants (1, 13, 30, 38, 45–47, 50).

Recently, a floating wetland system vegetated with aquatic plants has been used for water quality improvement and purification of various water resources such as stormwater, sewage, piggery effluent, poultry processing wastewater and water supply reservoirs (7, 14, 20, 44). In this system, water can be clarified and purified by the function of the plant roots and root microbes, while it directly passes the extensive roots hanging beneath the floating unit. To date, several previous studies have shown the important contribution of the root microbes for water quality improvement (45–47); however, there are few reports on microbes associated with the roots of aquatic plants including those grown in the floating treatment wetland system as well as natural environments.

Recently, we analyzed a root microbial community of a floating aquatic plant, *Spirodela polyrrhiza*, and found that it could be used to successfully isolate various novel microbes, including rarely cultivated organisms within the phylum *Verrucomicrobia*(28).

In the present study, we analyzed the microbial communities inhabiting the roots of two different emerged plants, reed (*Phagmites australis*) and Japanese loosestrife (*Lythrum anceps*), in the floating wetland system by culture-independent and culture-dependent approaches. The aims of this study are to obtain primary knowledge about the microbes associated with the roots of aquatic plants, and to verify whether the roots harbor unique microbial communities and are superior sources for culturing novel microbes.

## Materials and Methods

### Plant samples

Reed (*P. australis*) and Japanese loosestrife (*L. anceps*) were harvested from a pond (820 m^2^) located within Yamanashi prefectural wood park (Kanegawa-no-mori) in the summer (July 23, 2007). The pond receives water from Hirose Reservoir, which is fed by Fuefuki River located almost in Central Japan. These aquatic plants had been cultivated on a wooden floating base unit filled with fiber made from coconut husks for 5 years (from 2003 to 2007). The features of water chemistry in the pond such as nitrogen concentration, phosphorus concentration and temperature were described in our previous study (25).

### Culture-independent analysis

Total nucleic acids were extracted from 0.3 g (wet weight) of plant roots by using the ISOIL kit for bead beating (Nippon Gene, Tokyo, Japan). For DNA extraction from pond water (100 mL) collected from near the plants, the Ultraclean water DNA kit (MO BIO Laboratories, Carlsbad, CA, USA) was used. PCR amplification of the 16S rRNA genes from the extracted DNA was performed using two bacterial universal primers, EUB8F (48) (5’-AGAGTTTGATCMTGGCTCAG-3’: corresponding to positions 8–27 of the *Escherichia coli* 16S rRNA gene) and EUB1512R (19) (5’-ACGGYTACCTTGTTACGACTT-3’; corresponding to positions 1492–1512 of the *E. coli* 16S rRNA gene). The reactions were conducted as previously described (28) except that the numbers of cycles was reduced to minimize the PCR bias. The cycle number was adjusted to 18–25 cycles (18, 20, and 25 cycles for reed roots, Japanese loosestrife roots, and pond water, respectively). The amplified DNA fragments were purified by an illustra GFX PCR purification kit (GE Healthcare, Little Chalfont, UK), and cloned into the *E. coli* strain DH5α using the pT7 Blue T-vector kit (Novagen). The clonal DNA was amplified from randomly selected recombinants by colony direct PCR using the two primers, pT7-F (5’-GATCTACTAGTCATATGGAT-3’) and pT7-R (5’-TCGGTAC CCGGGGATCCGAT-3’), which were specific to the vector sites flanking the insert. The DNA fragments obtained were subjected to restriction fragment length polymorphism (RFLP) analysis by separate digestion with two different restriction endonucleases, *Hha*I and *Hae*III (Takara, Otsu, Japan). Coverage (*C*) values for each of the clone libraries were calculated by equation *C*=[1−(*n/N*)]×100 (9), where *n* is the number of unique clones and *N* is the total number of clones analyzed.

The PCR products from representative clones of each of the RFLP groups were purified with the GFX PCR DNA and Gel purification kit (GE Healthcare), and sequenced as previously described with primer EUB907R (41) (5’-CCGYCAATTCMTTT RAGTTT-3’). After checking the possible chimeric artifacts with the Bellerophon program (http://greengenes.lbl.gov/cgi-bin/nph-bel3_interface.cgi), the sequences were compared with those in the NCBI database using the BLASTn program (http://www.ncbi.nlm.nih.gov/blast/). Taxonomic classification of the clonal sequence at the level of the bacterial family was also conducted using the CLASSIFIER program (http://rdp.cme.msu.edu/classifier/classifier.jsp).

### Cultivation of microbes

A low-nutrient medium, DTS (pH 7.0), containing 0.17 g Bacto tryptone (Difco), 0.03 g Bacto soytone (Difco), 0.025 g glucose, 0.05 g NaCl and 0.025 g K_2_HPO_4_ in 1 L of distilled water, was used. Approximately 0.15 g (wet weight) of plant roots were gently rinsed twice with 30 mL sterilized DTS medium in a 50 mL test tube to remove microbes which were not associated with the plants, and the roots were mechanically homogenized with 10 mL DTS medium under the conditions at 15,000 rpm for 7 min by an Ace HOMOGENIZER AM-1 (Nihonseiki, Tokyo, Japan). The homogenates or pond water samples collected from an area surrounding the plants were diluted in 10-fold steps with DTS medium. Each diluted sample (50 μL) was independently inoculated on agar (1.5%) medium plates in triplicate, and incubated at 25°C for 30 days under dark conditions.

### Phylogenetic analysis of the isolates

16S rRNA genes of the isolated microbes were amplified by the colony direct PCR method using primers EUB8F and EUB1512R, and subjected to RFLP and sequencing analyses using methods similar to those described for culture-independent analysis with the exception that the restriction enzyme *Msp*I (Takara) was used in RFLP analysis instead of *Hae*III. The sequences were compared with those present in public databases using the BLASTn program. Taxonomic classification of the isolate at the family level was performed with the same method used for clone library analysis.

### Diversity index

The diversity of clones and isolated microbes at the level of the RFLP phylotype were calculated by the Shannon-Weiner index [(*H*)=−∑(*pi*) (ln *pi*)] and Simpson’s reciprocal index, 1/D, where D equals ∑(*pi*)^2^ and where *pi* is the proportion of clones or isolates within each phylotype *i* relative to the total number of clones or isolates.

### Sequencing of the 16S rRNA gene and phylogenetic analysis of strain YO-36

Phylogenetic analysis of strain YO-36 isolated was performed on the basis of 16S rRNA gene sequencing. The 16S rRNA gene of strain YO-36 was directly PCR-amplified with the universal primers 8F and 1492R (41) using AmpliTaq Gold (Applied Biosystems, Carlsbad, CA, USA). PCR was carried out in 100 μL reaction volumes in a Perkin-Elmer GeneAmp system 9700 (Perkin-Elmer Life Sciences, Boston, MA, USA) under the thermal cycle program as follows: initial denaturation at 95°C for 9 min, followed by 35 cycles of 95°C for 1 min, 50°C for 1 min, and 72°C for 2 min. The PCR product was purified with a MicroSpin S-400 HR column (GE Healthcare). Sequencing was performed with a CEQ DTCS-Quick Start kit (Beckman-Coulter, Fullerton, USA) and a CEQ-2000 automated sequence analyzer (Beckman). The sequence was compared with those from the public database using the BLASTn program. Phylogenetic analysis was performed with the ARB software package (23) using reference sequences with over 1,300 bp sequence length. After automatic and manual sequence alignments, a phylogenetic tree was constructed by the neighbor-joining (NJ) method as previously described (40). The bootstrap values were determined from 1,000 re-samplings with PAUP^*^ 4.0 (39) for the NJ method and TREEFINDER (17) for the maximum likelihood (ML) method as previously described (40).

### Nucleotide accession numbers

The GenBank/EMBL/DDBJ accession numbers for the 16S rRNA gene sequences of clones and isolates are AB540257–AB540432 and AB529661–AB529718, respectively.

## Results and Discussion

### Culture-independent analysis of microbes inhabiting roots of aquatic plants

**Fig. 1:**
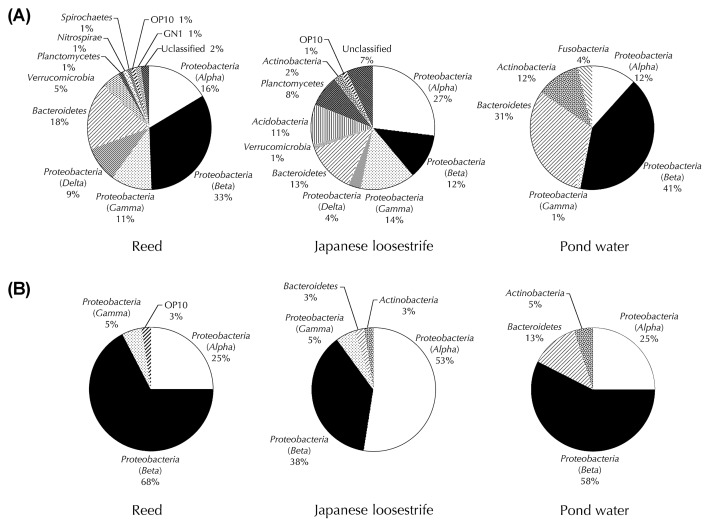
Phylogenetic distribution of the 16S rRNA gene clones (A) and isolates (B) belonging to different bacterial taxa in the roots of reed and Japanese loosestrife, and pond water.

Firstly, the microbial communities inhabiting the roots of reed and Japanese loosestrife were investigated using the 16S rRNA gene libraries of these two plants. For comparison, we also analyzed the microbial community in the surrounding environment, the pond water collected from near the aquatic plants. As shown in Supplementary Table S1, the clones (85 clones from each library; plastid- and mitochondria-derived sequences were not included) grouped on the basis of RFLP analysis could be divided into 66, 74 and 36 phylotypes (further referred to as C-phylotype; clone-phylotype), from clone libraries of reed, Japanese loosestrife and pond water, respectively. The coverage values were 32.9% for reed, 25.9% for Japanese loosestrife and 72.9% for pond water. Phyloge-netic analysis based on the sequencing of a clone representing each C-phylotype showed that the clone libraries of reed, Japanese loosestrife and pond water comprised of 11, 10 and 6 bacterial divisions (phylum or class (for *Proteobacteria*)), respectively (Fig. 1A). *Betaproteobacteria* was the most abundant bacterial division in reed (33% of total clones) and pond water samples (41%). In contrast, the most predominant division in Japanese loosestrife was *Alphaproteobacteria* (27%). Further taxonomic classification of the clonal sequences indicated that 64 clones from reed, 45 clones from Japanese loosestrife and 73 clones from pond water were placed within 22, 15 and 11 families, respectively (Table 1). Among 27 different families detected in the two aquatic plants, only 9 families (*Caulobacteraceae*, *Hyphomicrobiaceae*, *Rhodobacteraceae*, *Sphingomonadaceae*, *Comamonadaceae*, *Burkholderiales* genera incertae, *Chitinophagaceae*, *Saprospiraceae and Planctomycetaceae*) were shared in both samples. Furthermore, the most predominant bacterial families in the roots of reed and Japanese loosestrife were different; *Burkholderiales* genera incertae sedis for reed (15%) and *Sinobacteraceae* for Japanese loosestrife (12%). It has been reported that the bacterial community inhabiting the roots of terrestrial plants such as annual plants and trees apparently varies among different species (10, 11, 35, 36, 49). Therefore, the findings shown above suggested that aquatic plants might also harbor species-specific bacterial communities on the roots as is the case for terrestrial plants. We then compared the microbial community compositions between pond water and aquatic plant roots at the level of the bacterial family. The most predominant group in pond water was *Comamonadaceae* (38%), unlike those of two aquatic plants. Additionally, the predominant groups of the plant samples (*Burkholderiales* genera incertae sedis and *Sinobacteraceae*) were not detected in the pond water sample. This result indicated that the aquatic plant roots formed distinct microbial communities from the pond water.

To compare the microbial diversity among the two plant roots and the pond water, the Shannon-Weiner index and Simpson’s reciprocal index were calculated based on the grouping of C-phylotype (Table 2). Both the indices scores from two aquatic plants were higher than those from pond water, suggesting that the microbial communities of aquatic plant roots were more diverse than those of their surrounding pond water.

In the terrestrial environments, the microbial density and structures inhabiting the plant roots were distinct from those of root-free bulk soil (11, 26, 36). These phenomena are thought to be due to the distinct environments of the habitats formed by the surface structure and exudates released from the roots. The data obtained in the present study would also support this hypothesis in aquatic plants roots as well as in terrestrial plants.

**Fig. 2:**
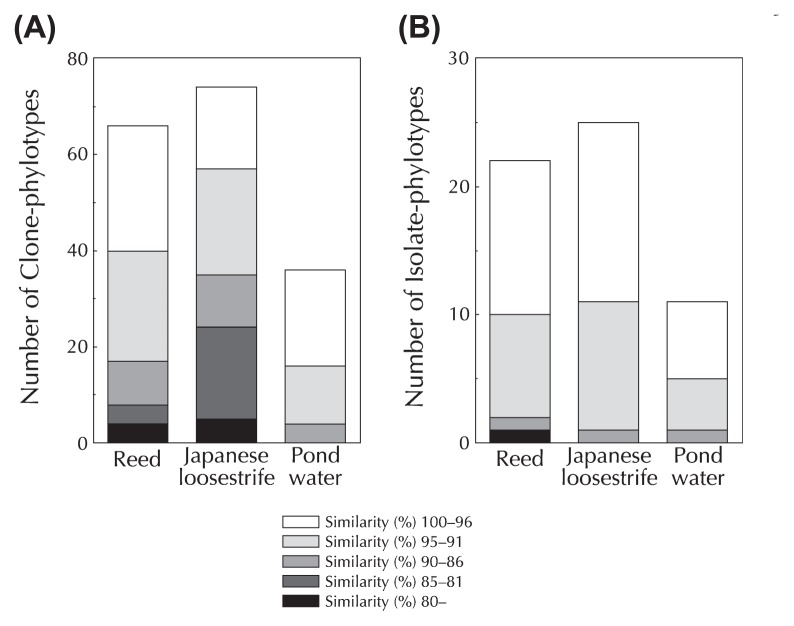
Novel clonal 16S rRNA gene sequences (A) and isolates (B) recovered from the roots of reed and Japanese loosestrife, and pond water. The similarity percentages between the clones (A) or isolates (B) and their closest species in the GenBank database are shown.

Some candidate phyla and rarely cultured groups such as the *Verrucomicrobia* and *Acidobacteria* were found in the clone libraries of roots of two aquatic plants. The *Verrucomicrobia* and candidate phylum OP10 (recently named the phylum *Armatimonadetes*(42); see below) were detected in both aquatic plants. Candidate phylum GN1 and *Acidobacteria* were also found in reed and Japanese loosestrife rhizospheres (Fig. 1A, Table S1); however, these phyla were not detected in the clone library of pond water (Fig. 1A, Table S1). Furthermore, unclassifiable bacterial sequences (*i.e.*, C-phylotypes Nos. 26 and 35 from reed and Nos. 70, 78, 131 and 134 from Japanese loosestrife) were found in the clone libraries of the two aquatic plants, but not in pond water (Fig. 1A, Table S1). When clones with sequences with less than 95% similarity to the 16S rRNA gene of any known bacterial species were regarded as phylogenetically novel microbes, then more C-phylotypes from the roots of aquatic plants showed phylogenetic novelty (40 C-phylotypes for reed and 57 C-phylotypes for Japanese loosestrife), compared to pond water (16 C-phylotypes) (Fig. 2A). In addition, C-phylotypes showing less than 90% sequence similarity to any known bacterial species were detected in both reed and Japanese loosestrife rhizospheres, but not in pond water (Fig. 2A). These results clearly indicated that the roots of aquatic plants harbor far more novel organisms than pond water.

### Cultivation and isolation of microbes from roots of aquatic plants

To verify whether novel organisms can be cultured from the roots of aquatic plants, microbial cultivation and isolation experiments were conducted. Homogenates of reed roots and Japanese loosestrife roots, and pond water were independently inoculated on low-nutrient medium plates and incubated at 25°C. The numbers of visible colonies continuously increased for more than 20 days. After 30 days of cultivation, maximum viable counts of 4.0×10^8^±2.3×10^6^ CFU g^−1^ (wet weight), 1.1×10^8^±1.7×10^7^ CFU g^−1^ (wet weight) and 9.7× 10^5^±1.9×10^5^ CFU mL^−1^ were obtained from reed, Japanese loosestrife, and pond water, respectively. Forty colonies were randomly selected from the medium plates with appropriate dilution (approx. 50 to 80 colonies on a plate), and their 16S rRNA genes were PCR-amplified and subjected to RFLP analysis. The isolates from the two plants were grouped into 45 phylotypes (hereafter referred to as I-phylotype (meaning isolate-phylotype) numbered 1 to 45) consisting of 22 for reed and 25 for Japanese loosestrife. By contrast, the isolates from pond water consisted of 11 I-phylotypes numbered 46 to 56 (Table 3). The phylogenetic relationships of the isolates and their closely related species were analyzed on the basis of partial 16S rRNA gene sequences (517 to 712 bp). The 16S rRNA gene sequences of the representative isolates for each I-phylotype were determined and compared with the sequences in the NCBI database. The most closely related species of the isolates are shown in Table 3. *Betaproteobacteria* and *Alphaproteobacteria* were the most abundant bacterial divisions in cultivation studies of reed roots and pond water, and in Japanese loosestrife roots, respectively, similar to the pattern in culture-independent analysis (Fig. 1B). In further taxonomic classification at the family level, the isolates from reed (33 isolates), Japanese loosestrife (32 isolates) and pond water (37 isolates) were divided into 7, 11 and 8 groups, respectively. The predominant bacterial families in reed and pond water were *Burkholderiales* genera incertae sedis for reed (47.5%) for reed and *Comamonadaceae* (40%) for pond water as found in culture-independent analysis; however, the predominant family in Japanese loosestrife was different between culture-dependent (*Alcaligenaceae*; 20%) and culture-independent (*Sinobacteraceae*; 12%) methods as described in previous studies in a wide variety of natural habitats (5, 6, 18, 21, 31).

The scores of diversity indices based on the grouping of I-phylotypes indicated that the isolates obtained from two aquatic plant roots were more diverse than from pond water (Table 2). These results may be due to the diverse niche-associated occurrence of microbial communities on aquatic plant roots, as shown in culture-independent analysis.

Twenty I-phylotypes showing below 95% 16S rRNA gene sequence similarity (a criterion indicating novel microbes defined in culture-independent analysis; see above) to validly described species were obtained from two aquatic plants (10 I-phylotypes from reed and 11 I-phylotypes from Japanese loosestrife). By contrast, only 5 I-phylotypes showing phylogenetic novelty were recovered from pond water (Table 3, Fig. 2B). This also suggested that aquatic plants harbor more readily-cultured novel microbes in their root than their surrounding water environments.

Most importantly, we succeeded in isolating a novel microorganism, strain YO-36 (I-phylotype No. 19), from the reed roots. Phylogenetic analysis based on 16S rRNA gene sequence (almost full length, >1,300 bp) clearly revealed that strain YO-36 belongs to the candidate phylum OP10 that includes clones CYO-76 (C-phylotype No. 62 from reed) and CMI-72 (C-phylotype No. 127 from Japanese loosestrife) obtained by direct molecular analysis of the samples. The highest sequence similarity of YO-36 was to clone CYO-76 (98.5%) and a previously described environmental clone (clone 111ds10 obtained from manure-discharged water) (34) (97.7%), whereas the identity with clone CMI-72 was fairly low (81.4%). Strain YO-36 showed significantly low sequence similarities (<80%) to any known validly described species but shared 90–91% similarities with some environmental clonal sequences derived from members of the candidate phylum OP10 (4, 22) (Table 3). Therefore, very recently, based on polyphasic taxonomic analyses (morphological, physiological and phylogenetic characterizations), we proposed a novel genus and species for this particular strain YO-36, *Armatimonas rosea* gen. nov., sp. nov., and also proposed a new phylum *Armatimonadetes* for the candidate phylum OP10 and a new class *Armatimonadia* within the phylum (42).

### Concluding remarks

In this study, we clearly showed that the roots of aquatic plants harbor unique, diverse, and novel microbes. Moreover, we also succeeded in the cultivation and isolation of a diverse array of phylogenetically novel microbes (20 I-phylotypes from the roots), including an isolate within the candidate phylum OP10 by employing relatively long cultivation (30 days) using a low-nutrient agar plate medium that does not require any unique reagents, organisms and materials that have been used for the isolation of rarely cultivated groups in other studies (2, 3, 12, 15, 16, 27, 29, 33, 37, 40, 43, 51). In our previous study, a variety of novel microbes belonging to the rarely cultured phylum *Verrucomirobia* were successfully isolated from the roots of a floating aquatic plant, *S. polyrrhiza*, with no vigorous efforts, as was the case in this study. Together with the previous findings, the present study clearly demonstrated that the roots of aquatic plants can be highly intriguing sources of novel organisms. Functional analyses of these organisms would provide further insights into the interactions occurring between microbes and aquatic plants. Further study will also reveal the microbial community structure and diversity in aquatic plant roots from the natural environment as well as those from the floating treatment wetland system via advanced techniques with 16S pyrosequencing technology.

## Supporting information



## Figures and Tables

**Fig. 3:**
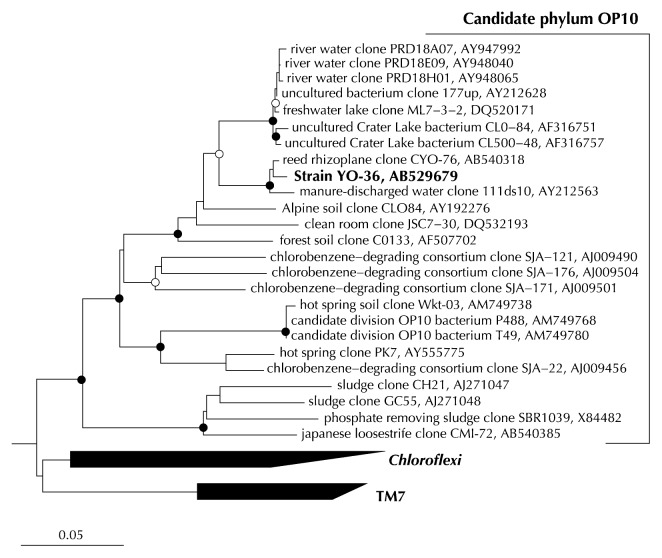
Phylogenetic tree showing the relationship between strain YO-36 and related sequences of the candidate phylum OP10. The tree was constructed using the neighbor-joining (NJ) method on the basis of the 16S rRNA gene sequences. Node with bootstrap values of >90% and >80%, estimated using the NJ method and maximum likelihood (ML) methods, are shown as a closed circle and an open circle, respectively. The tree was rooted against a group of 26 sequences belonging to different phyla in the domains *Bacteria* and *Archaea*. Scale bar indicates 0.05 substitutions per nucleotide position.

**Table 1:** Taxonomic classification of clones and isolates

Phylum	Class	Order	Family	Number of clones			Number of isolates		
	
				Reed	Japanese loosestrife	Pond water	Reed	Japanese loosestrife	Pond water
*Proteobacteria*	*Alphaproteobacteria*	*Caulobacterales*	*Caulobacteraceae*	1	1			3	
		*Rhizobiales*	*Beijerinckiaceae*		2				
			*Bradyrhizobiaceae*		1			1	
			*Hyphomicrobiaceae*	1	1			1	
			*Rhizobiaceae*				1		
			*Xanthobacteraceae*					1	
			Unclassified	1	8	1	2	7	
		*Rhodobacterales*	*Rhodobacteraceae*	4	2	9	3	4	9
		*Sphingomonadales*	*Erythrobacteraceae*		1				
			*Sphingomonadaceae*	1	5		4	4	1
			Unclassified	1					
		Unclassified	Unclassified	6	2				

	*Betaproteobacteria*	*Burkholderiales*	*Alcaligenaceae*		2			8	
			*Burkholderiaceae*			2			1
			*Comamonadaceae*	7	1	32	4	3	16
			*Oxalobacteraceae*	1					5
			*Burkholderiales* genera incertae sedis	13	2		19	4	
			Unclassified				1		
		*Methylophilales*	*Methylophilaceae*		4	1			1
		*Rhodocyclales*	*Rhodocyclaceae*	7			1		
		Unclassified	Unclassified		1		2		

	*Deltaproteobacteria*	*Bdellovibrionales*	*Bdellovibrionaceae*	1					
		*Myxococcales*	*Cystobacterineae*	1					
			*Polyangiaceae*	1					
			Unclassified	4	2				
		Unclassified	Unclassified		1				

	*Gammaproteobacteria*	*Legionellales*	*Legionellaceae*			1			
		*Methylococcales*	*Methylococcaceae*	2					
		*Pseudomonadales*	*Pseudomonadaceae*	6			1		
		*Xanthomonadales*	*Sinobacteraceae*		10			2	
		Unclassified	Unclassified	1	2		1		

*Bacteroidetes*	*Flavobacteria*	*Flavobacteriales*	*Cryomorphaceae*	5		2			
			*Flavobacteriaceae*	2		8			3
	*Sphingobacteria*	*Sphingobacteriales*	*Chitinophagaceae*	2	6	2		1	1
			*Cytophagaceae*	2		11			
			*Saprospiraceae*	1	2				
			Unclassified	1	2				
	Unclassified	Unclassified	Unclassified	2	1	3			1

*Verrucomicrobia*	*Opitutae*	*Opitutales*	*Opitutaceae*	3					
	Subdivision 3	Unclassified	Unclassified	1	1				

*Acidobacteria*	*Acidobacteria* Gp3	Unclassified	Unclassified		6				
	*Acidobacteria* Gp4	Unclassified	Unclassified		2				
	*Acidobacteria* Gp6	Unclassified	Unclassified		1				

*Planctomycetes*	*Planctomycetacia*	*Planctomycetales*	*Planctomycetaceae*	1	5				
	Unclassified	Unclassified	Unclassified		2				

*Fusobacteria*	*Fusobacteria*	*Fusobacteriales*	*Fusobacteriaceae*			3			

*Nitrospira*	*Nitrospira*	*Nitrospirales*	*Nitrospiraceae*	1					

*Spirochaetes*	*Spirochaetes*	*Spirochaetales*	*Spirochaetaceae*	1					

*Actinobacteria*	*Actinobacteria*	*Actinomycetales*	*Microbacteriaceae*			2			
			Unclassified			8		1	2
		Unclassified	Unclassified		1				
	Unclassified	Unclassified	Unclassified		1				

Candidate phylum OP10	Unclassified	Unclassified	Unclassified	1	1		1		

Candidate phylum GN1	Unclassified	Unclassified	Unclassified	1					

Unclassified	Unclassified	Unclassified	Unclassified	2	6				

Total analyzed clone or isolate numbers				85	85	85	40	40	40
Total numbers of clones or isolates classified at the family level				64	45	73	33	32	37

**Table 2:** Diversity indices for clones and isolates at the phylotype level

Sample	Clone		Isolate	
	
	Shannon-Weiner index	Simpson’s reciprocal index	Shannon-Weiner index	Simpson’s reciprocal index
Reed roots	4.04	45.44	2.82	12.90
Japanese loosestrife roots	4.26	67.52	2.96	13.56
Pond water	3.15	15.67	2.04	6.30

**Table 3:** Isolated microbes in this study and their related authentic species on the basis of 16S rRNA gene sequence

Isolate- phylotype No.^a^	Isolate						Authentic species (Accession No.)	Identity (%)	Phylum (Class)	Length (bp)

	Reed		Japanese loosestrife		Pond water					
		
	Total no. of isolates	Name of representative strain	Total no. of isolates	Name of representative strain	Total no. of isolates	Name of representative strain				
1	5	YO-23					*Rubrivivax gelatinosus* strain ATCC17011 (D16213)	97	*Proteobacteria (Beta)*	709
2	6	YO-32	1	MI-15			*Aquincola tertiaricarbonis* strain L10^T^ (DQ656489)	97	*Proteobacteria (Beta)*	711
3	5	YO-28					*Methylibium fulvum* strain Gsoil 328 (AB245357)	97	*Proteobacteria (Beta)*	653
4	1	YO-5					*Azoarcus buckelii* strain U120 (AJ315676)	94	*Proteobacteria (Beta)*	711
5	3	YO-6					*Curvibacter delicatus* strain LMG 4328 (AF078756)	97	*Proteobacteria (Beta)*	696
6	1	YO-8	1	MI-36			*Chelatovorus multitrophus* strain DSM 9103^T^ (EF457243)	91	*Proteobacteria (Alpha)*	709
7	1	YO-9					*Herbaspirillum seropedicae* strain ATCC 35892 (Y10146)	91	*Proteobacteria (Beta)*	710
8	1	YO-13					*Bosea thiooxidans* strain BI-42 (AF508803)	93	*Proteobacteria (Alpha)*	710
9	1	YO-14					*Rhodobacter ovatus* strain JA234^T^ (AM690348)	98	*Proteobacteria (Alpha)*	709
10	1	YO-16					*Limnobacter thiooxidans* strain CS-K2 (AJ289885)	91	*Proteobacteria (Beta)*	697
11	1	YO-17					*Sandaracinobacter sibiricus* strain RB16-17 (Y10678)	97	*Proteobacteria (Alpha)*	710
12	1	YO-18					*Rhodobacter blasticus* strain ATCC 33485^T^ (DQ342322)	98	*Proteobacteria (Alpha)*	709
13	3	YO-19					*Ideonella dechloratans* (X72724)	98	*Proteobacteria (Beta)*	710
14	1	YO-26					*Leptothrix discophora* strain SS-1 (L33975)	95	*Proteobacteria (Beta)*	517
15	1	YO-27					*Pseudomonas pohangensis* strain H3-R18^T^ (DQ339144)	98	*Proteobacteria (Gamma)*	711
16	1	YO-29					*Paracoccus marcusii* (Y12703)	95	*Proteobacteria (Alpha)*	709
17	1	YO-33					*Nitrosococcus oceani* strain ATCC 19707 (AY690336)	87	*Proteobacteria (Gamma)*	708
18	1	YO-34					*Pelomonas aquatica* strain CCUG 52575^T^ (AM501435)	97	*Proteobacteria (Beta)*	695
19	1	YO-36					*Desulfotomaculum halophilum* strain SEBR 3139 (U88891; *Fir-micutes*)	80	*Candidate phylum OP10*	709
20	1	YO-38					*Novosphingobium hassiacum* strain W-51 (AJ416411)	97	*Proteobacteria (Alpha)*	711
21	1	YO-40					*Sinorhizobium adhaerens* strain 5D19 (AJ420773)	98	*Proteobacteria (Alpha)*	709
22	2	YO-45					*Novosphingobium resinovorum* strain NCIMB 8767 (EF029110)	94	*Proteobacteria (Alpha)*	712
23			8	MI-1			*Azohydromonas lata* strain IAM 12665 (AB201626)	97	*Proteobacteria (Beta)*	690
24			3	MI-2			*Rubrivivax gelatinosus* strain ATCC 17011 (D16213)	96	*Proteobacteria (Beta)*	696
25			1	MI-5			*Sphingobium japonicum* strain UT26 (AF039168)	97	*Proteobacteria (Alpha)*	712
26			1	MI-6			*Rhodobacter blasticus* strain ATCC 33485^T^ (DQ342322)	98	*Proteobacteria (Alpha)*	712
27			1	MI-8			*Steroidobacter denitrificans* strain FS (EF605262)	93	*Proteobacteria (Gamma)*	707
28			2	MI-9			*Rhodobacter capsulatus* strain ATCC 11166^T^ (DQ342320)	96	*Proteobacteria (Alpha)*	695
29			1	MI-10			*Niastella jeongjuensis* strain GR20-13 (DQ244076)	94	*Bacteroidetes*	710
30			1	MI-11			*Asticcacaulis biprosthecium* strain DSM 4723^T^ (AJ247193)	98	*Proteobacteria (Alpha)*	710
31			1	MI-12			*Phenylobacterium lituiforme* strain FaiI3 (AY534887)	97	*Proteobacteria (Alpha)*	711
32			1	MI-13			*Novosphingobium hassiacum* strain W-51 (AJ416411)	97	*Proteobacteria (Alpha)*	711
33			3	MI-14			*Rhodoferax antarcticus* strain Fryx1 (AY609198)	98	*Proteobacteria (Beta)*	696
34			3	MI-16			*Hyphomicrobium hollandicum* strain IFAM KB-677 (Y14303)	93	*Proteobacteria (Alpha)*	660
35			1	MI-20			*Sphingomonas suberifaciens* strain IFO 15211 (D13737)	95	*Proteobacteria (Alpha)*	709
36			1	MI-26			*Caulobacter henricii* strain ATCC 15253^T^ (AJ227758)	98	*Proteobacteria (Alpha)*	708
37			1	MI-30			*Methylosinus trichosporium* strain NCIMB 11131 (Y18947)	91	*Proteobacteria (Alpha)*	709
38			1	MI-31			*Novosphingobium resinovorum* strain NCIMB 8767^T^ (EF029110)	96	*Proteobacteria (Alpha)*	709
39			1	MI-32			*Pseudodevosia insulae* strain DS- 56^T^ (EF012357)	96	*Proteobacteria (Alpha)*	711
40			1	MI-33			*Acidimicrobium ferrooxidans* strain DSM 10331 (AFU75647)	90	*Actinobacteria*	712
41			1	MI-34			*Rhodopseudomonas cryptolactis* strain DSM 9987 (AB087718)	95	*Proteobacteria (Alpha)*	712
42			1	MI-35			*Bradyrhizobium elkanii* strain USDA 76 (U35000)	99	*Proteobacteria (Alpha)*	711
43			1	MI-37			*Steroidobacter denitrificans* strain FS (EF605262)	93	*Proteobacteria (Gamma)*	709
44			2	MI-40			*Devosia insulae* strain DS-56^T^ (EF012357)	92	*Proteobacteria (Alpha)*	712
45			1	MI-39			*Afipia massiliensis* strain CCUG 45153^T^ (AY029562)	93	*Proteobacteria (Alpha)*	710
46					9	KW-15	*Rhodobacter massiliensis* strain Framboise (AF452106)	98	*Proteobacteria (Alpha)*	709
47					3	KW-13	*Flavobacterium terrae* strain R2A1-13^T^ (EF117329)	94	*Bacteroidetes*	711
48					9	KW-16	*Limnohabitans curvus* strain MWH-C5 (AJ938026)	98	*Proteobacteria (Beta)*	710
49					7	KW-29	*Limnohabitans curvus* strain MWH-C5 (AJ938026)	97	*Proteobacteria (Beta)*	710
50					5	KW-28	*Herbaspirillum rubrisubalbicans* strain M4 (AJ238356)	97	*Proteobacteria (Beta)*	710
51					1	KW-22	*Novosphingobium aromati-civorans* strain SMCC B0695 (U20755)	98	*Proteobacteria (Alpha)*	711
52					1	KW-39	*Pedobacter caeni* strain R-21937	86	*Bacteroidetes*	711
53					1	KW-40	*Methylotenera mobila* strain JLW8^T^ (DQ287786)	94	*Proteobacteria (Beta)*	712
54					2	KW-45	*Tetrasphaera nostocoidensis* strain Ben 70 (DQ007320)	91	*Actinobacteria*	709
55					1	KW-42	*Polynucleobacter necessarius* strain STIR1 (AJ811014)	99	*Proteobacteria (Beta)*	682
56					1	KW-43	*Chitinophaga ginsengisegetis* strain Gsoil 040^T^ (AB264798)	91	*Bacteroidetes*	680

Total			40		40					

Novel microbes^b^			14 (11)		8 (5)					

a The phylotypes were defined on the basis of the results of restriction fragment length polymorphism (RFLP) analysis. The isolate-phylotypes whose sequences indicated less than 95% identity with those from authentic species are underlined.

b The number of phylotypes showing phylogenetic novelty is shown in parentheses.
