# Supplementary material for: Microbial Community Analysis in the Roots of Aquatic Plants and Isolation of Novel Microbes Including an Organism of the Candidate Phylum OP10
Source: Microbes Environ. 2011 Dec 6;27(2):149–57. doi: 10.1264/jsme2.ME11288 (PMC4036017; doi:10.1264/jsme2.ME11288)
Supplement: Supplementary file 1 [file 27_149_s1.pdf]

Supporting information

Supplementary Table S1. Phylogenetic affiliations of 16S rRNA gene clones

Clone-phylotype No.[a]	Reed		Japanese loosestrife		Pond water		Authentic species (Accession No.)	Identity (%)	Phylum (Class)	Length (bp)
	Total no. of clone	Name of representative clone	Total no. of clone	Name of representative clone	Total no. of clone	Name of representative clone				
1	6	CYO-1					*Cellvibrio mixtus subsp. mixtus* strain ACM 2601 (AF448515)	94	*Proteobacteria (Gamma)*	553
2	1	CYO-2					*Flavobacterium segetis* strain AT1048^T (AY581115)	96	*Bacteroidetes*	591
3	1	CYO-3					*Leptothrix discophora* strain SP-6 (L33974)	97	*Proteobacteria (Beta)*	646
4	2	CYO-4			13	CKW-13	*Limnohabitans curvus* strain MWH-C5 (AJ938026)	97	*Proteobacteria (Beta)*	514
5	2	CYO-5					*Roseateles aquatilis* strain CCUG48205^T (AM501446)	97	*Proteobacteria (Beta)*	568
6	1	CYO-6					*Aquaspirillum delicatum* strain LMG 4328 (AF078756)	97	*Proteobacteria (Beta)*	523
7	2	CYO-7					*Leptothrix cholodnii* strain CCM 1827 (X97070)	97	*Proteobacteria (Beta)*	620
8	1	CYO-8					*Aquaspirillum delicatum* strain LMG 4328 (AF078756)	98	*Proteobacteria (Beta)*	607
9	1	CYO-9					*Rhodoferax ferrireducens* strain T118^T (CP000267)	98	*Proteobacteria (Beta)*	579
10	4	CYO-10					*Azohydromonas lata* strain IAM 12665 (AB201626)	97	*Proteobacteria (Beta)*	581
11	1	CYO-11					*Rhodoferax antarcticus* strain Fryx1 (AY609198)	98	*Proteobacteria (Beta)*	567
12	1	CYO-12			2	CKW-28	*Aquaspirillum delicatum* strain LMG 4328 (AF078756)	98	*Proteobacteria (Beta)*	604
13	1	CYO-13					*Prosthecobacter debontii* strain DSM 14044 (AJ966882)	82	*Verrucomicrobia*	536
14	1	CYO-14					*Spirochaeta aurantia* strain J1 (M57740)	97	*Spirochaetes*	591

Supplementary Table S1. (*continued*)

Clone-phylotype No.[a]	Reed		Japanese loosestrife		Pond water		Authentic species (Accession No.)	Identity (%)	Phylum (Class)	Length (bp)
	Total no. of clone	Name of representative clone	Total no. of clone	Name of representative clone	Total no. of clone	Name of representative clone				
15	1	CYO-15					*Ideonella dechloratans* (X72724)	98	*Proteobacteria (Beta)*	569
16	1	CYO-17					*Owenweeksia hongkongensis* strain UST20020801 (AB125062)	88	*Bacteroidetes*	530
17	1	CYO-18					*Pedobacter terricola* strain DS-45 (EF446147)	82	*Bacteroidetes*	571
18	1	CYO-19					*Lewinella agarilytica* strain SST-19 (AM286229)	86	*Bacteroidetes*	566
19	1	CYO-20					*Methylobacter tundripaludum* strain SV96^{T} (AJ414655)	91	*Proteobacteria (Gamma)*	525
20	3	CYO-22					*Rhodobacter blasticus* strain ATCC 33485^{T} (DQ342322)	98	*Proteobacteria (Alpha)*	585
21	1	CYO-23	2	CMI-5	1	CKW-12	*Sediminibacterium salmoneum* strain NJ-44 (EF407879)	97	*Bacteroidetes*	598
22	1	CYO-25			1	CKW-25	*Labrys miyagiensis* strain G24116 (AB236171)	92	*Proteobacteria (Alpha)*	556
23	1	CYO-26					*Blastochloris sulfoviridis* strain DSM729 (D86514)	87	*Proteobacteria (Alpha)*	357
24	1	CYO-27					*Asticcacaulis biprosthecium* strain DSM 4723^{T}	95	*Proteobacteria (Alpha)*	306
25	1	CYO-28					*Azonexus fungiphilus* strain BS5-8 (AF011350)	97	*Proteobacteria (Beta)*	539
26	1	CYO-29					*Ehrlichia muris* strain AS145 (U15527; *Proteobacteria*)	75	Unclassified	611
27	1	CYO-30					*Emticicia oligotropha* strain GPTSA100-15 (AY904352)	94	*Bacteroidetes*	649
28	1	CYO-31					*Emticicia oligotropha* strain GPTSA100-15 (AY904352)	94	*Bacteroidetes*	519
29	1	CYO-32					*Ochrobactrum anthropi* strain ATCC 49188 (CP000759)	90	*Proteobacteria (Alpha)*	598
30	1	CYO-36					*Azospirillum amazonense* strain DSM 2787 (Z29616)	92	*Proteobacteria (Alpha)*	640

Supplementary Table S1. (*continued*)

Clone-phylotype No.[a]	Reed		Japanese loosestrife		Pond water		Authentic species (Accession No.)	Identity (%)	Phylum (Class)	Length (bp)
	Total no. of clone	Name of representative clone	Total no. of clone	Name of representative clone	Total no. of clone	Name of representative clone				
31	1	CYO-37					*Rubrivivax gelatinosus* strain ATCC17011 (D16213)	98	*Proteobacteria (Beta)*	528
32	1	CYO-38					*Pseudodevosia insulae* strain DS-56 (EF012357)	98	*Proteobacteria (Alpha)*	556
33	1	CYO-40					*Sphingomonas xenophaga* strain BN6 (X94098)	96	*Proteobacteria (Alpha)*	574
34	1	CYO-41					*Dechloromonas hortensis* strain MA-1 (AY277621)	98	*Proteobacteria (Beta)*	574
35	1	CYO-42					*Persephonella guaymasensis* strain H2 (AF385630; *Aquificales*)	76	Unclassified	568
36	4	CYO-43					*Fluviicola taffensis* strain RW262 (AF493694)	94	*Bacteroidetes*	812
37	1	CYO-44					*Phenylobacterium koreense* strain Slu-01 (AB166881)	89	*Proteobacteria (Alpha)*	573
38	3	CYO-45					*Denitratisoma oestradiolicum* strain AcBE2-1 (AY879297)	95	*Proteobacteria (Beta)*	565
39	1	CYO-48					*Undibacterium pigrum* strain CCUG 49009 (AM397630)	98	*Proteobacteria (Beta)*	532
40	2	CYO-49					*Rubrivivax gelatinosus* strain ATCC17011 (D16213)	98	*Proteobacteria (Beta)*	533
41	1	CYO-50					*Methylobacter tundripaludum* strain SV96^{T} (AJ414655)	93	*Proteobacteria (Gamma)*	596
42	1	CYO-51					*Nitrospira moscoviensis* (X82558)	98	*Nitrospirae*	571
43	1	CYO-52					*Rhodobium orientis* strain MB312 (D30792)	87	*Proteobacteria (Alpha)*	603
44	1	CYO-53					*Flexibacter ruber* strain IFO 16675 (AB078064)	85	*Bacteroidetes*	620
45	1	CYO-54					*Bdellovibrio bacteriovorus* strain DSM 50705 (AJ278145)	92	*Proteobacteria (Delta)*	463
46	1	CYO-55					*Terrimonas lutea* strain 14990Y (AB192292)	93	*Bacteroidetes*	646
47	1	CYO-56					*Opitutus terrae* strain ACB90 (AJ229246)	91	*Verrucomicrobia*	642

Supplementary Table S1. (*continued*)

Clone-phylotype No.[a]	Reed		Japanese loosestrife		Pond water		Authentic species (Accession No.)	Identity (%)	Phylum (Class)	Length (bp)
	Total no. of clone	Name of representative clone	Total no. of clone	Name of representative clone	Total no. of clone	Name of representative clone				
48	1	CYO-57					*Byssophaga cruenta* strain DSM 14553^{T} (AJ833647)	95	*Proteobacteria (Delta)*	646
49	1	CYO-58					*Opitutus terrae* strain ACB90 (AJ229246)	92	*Verrucomicrobia*	596
50	1	CYO-59					*Thermosulfidibacter takaii* strain ABI70S6 (AB282756; *Aquificales*)	78	Candidate phylum GN1	619
51	1	CYO-60					*Acidithiobacillus ferrooxidans* strain DSM 2392 (AJ459800)	86	*Proteobacteria (Gamma)*	647
52	1	CYO-62	1	CMI-18			*Haliscomenobacter hydrossis* strain DSM 1100 (EF554367)	86	*Bacteroidetes*	620
53	1	CYO-63					*Cystobacter violaceus* strain Cb vi29 (AJ233905)	99	*Proteobacteria (Delta)*	523
54	1	CYO-64					*Opitutus terrae* strain ACB90 (AJ229246)	92	*Verrucomicrobia*	640
55	1	CYO-65					*Chondromyces lanuginosus* strain Sy t2 (AJ233939)	91	*Proteobacteria (Delta)*	645
56	1	CYO-66					*Paracoccus aminovorans* strain JCM 7685 (D32240)	97	*Proteobacteria (Alpha)*	610
57	1	CYO-68					*Polyangium thaxteri* strain Pl t3 (AJ233943)	91	*Proteobacteria (Delta)*	814
58	1	CYO-69					*Chondromyces apiculatus* strain Cm a14 (AJ233938)	92	*Proteobacteria (Delta)*	694
59	1	CYO-71					*Dechloromonas hortensis* strain MA-1 (AY277621)	98	*Proteobacteria (Beta)*	804
60	1	CYO-73					*Trojanella thessalonices* strain L13 (AF069496)	89	*Proteobacteria (Alpha)*	799
61	1	CYO-74					*Gemmata obscuriglobus* strain ACM 2246	85	*Planctomycetes*	780
62	1	CYO-76					*Curtobacterium ammoniigenes* strain NBRC 101789 (AB266600; *Actinobacteria*)	79	Candidate phylum OP10	807

Supplementary Table S1. (*continued*)

Clone-phylotype No.[a]	Reed		Japanese loosestrife		Pond water		Authentic species (Accession No.)	Identity (%)	Phylum (Class)	Length (bp)
	Total no. of clone	Name of representative clone	Total no. of clone	Name of representative clone	Total no. of clone	Name of representative clone				
63	1	CYO-77			8	CKW-22	*Flavobacterium terrae* strain R2A1-13^T (EF117329)	94	*Bacteroidetes*	640
64	1	CYO-78					*Polyangium thaxteri* strain Pl t3 (AJ233943)	91	*Proteobacteria (Delta)*	735
65	1	CYO-79					*Fluviicola taffensis* strain RW262 (AF493694)	94	*Proteobacteria (Delta)*	803
66	1	CYO-80					*Dechloromonas hortensis* strain MA-1 (AY277621)	95	*Proteobacteria (Beta)*	817
67			1	CMI-1			*Methylophilus methylotrophus* strain NCIMB 10515 (AB193724)	95	*Proteobacteria (Beta)*	619
68			1	CMI-2			*Methylophilus methylotrophus* strain NCIMB 10515 (AB193724)	97	*Proteobacteria (Beta)*	656
69			1	CMI-3			*Beijerinckia mobilis* strain DSM 2326 (AJ563932)	93	*Proteobacteria (Alpha)*	659
70			2	CMI-4			*Bacteroides capillosus* strain ATCC 29799 (AY136666; *Bacteroidetes*)	84	Unclassified	625
71			2	CMI-6			*Steroidobacter denitrificans* strain FS (EF605262)	93	*Proteobacteria (Gamma)*	539
72			1	CMI-7			*Steroidobacter denitrificans* strain FS (EF605262)	94	*Proteobacteria (Gamma)*	582
73			1	CMI-8			*Clostridium putrefaciens* strain DSM 1291 (AF127024; *Firmicutes*)	83	*Acidobacteria*	640
74			1	CMI-9			*Flavisolibacter ginsengisoli* strain Gsoil 643 (AB267477)	94	*Bacteroidetes*	651
75			1	CMI-10	9	CKW-3	*Haematobacter massiliensis* strain CCUG 47968 (DQ342309)	98	*Proteobacteria (Alpha)*	557
76			1	CMI-11			*Steroidobacter denitrificans* strain FS (EF605262)	93	*Proteobacteria (Gamma)*	600
77			1	CMI-12			*Lachnobacterium bovis* strain LRC 5436 (AF298665; *Firmicutes*)	80	*Acidobacteria*	625
78			2	CMI-13			*Bacteroides capillosus* strain ATCC 29799 (AY136666; *Bacteroidetes*)	84	Unclassified	640

Supplementary Table S1. (*continued*)

Clone-phylotype No.[a]	Reed		Japanese loosestrife		Pond water		Authentic species (Accession No.)	Identity (%)	Phylum (Class)	Length (bp)
	Total no. of clone	Name of representative clone	Total no. of clone	Name of representative clone	Total no. of clone	Name of representative clone				
79			1	CMI-14			*Haliscomenobacter hydrossis* strain DSM 1100 (AJ784892; *Bacteroidetes*)	85	*Bacteroidetes*	590
80			2	CMI-15			*Steroidobacter denitrificans* strain FS (EF605262)	92	*Proteobacteria (Gamma)*	556
81			1	CMI-16			*Clostridium aminophilum* strain F (L04165; *Firmicutes*)	82	*Acidobacteria*	630
82			2	CMI-19			*Azohydromonas lata* strain IAM 12665 (AB201626)	98	*Proteobacteria (Beta)*	578
83			1	CMI-20			*Rhodoferax ferrireducens* strain T118 (AF435948)	98	*Proteobacteria (Beta)*	591
84			1	CMI-21			*Clostridium aminophilum* strain F (L04165; *Firmicutes*)	82	*Acidobacteria*	520
85			1	CMI-22			*Clostridium aminophilum* strain F (L04165; *Firmicutes*)	83	*Acidobacteria*	640
86			1	CMI-23			*Prosthecobacter debontii* strain DSM 14044 (AJ966882)	84	*Verrucomicrobia*	633
87			2	CMI-24			*Aquincola tertiaricarbonis* strain L10 (DQ656489)	95	*Proteobacteria (Beta)*	653
88			1	CMI-25			*Aquaspirillum itersonii subsp. nipponicum* strain LMG 7370 (EF612766)	88	*Proteobacteria (Alpha)*	558
89			1	CMI-29			*Hyphomicrobium sulfonivorans* strain ATCC BAA-113 (AF235089)	96	*Proteobacteria (Alpha)*	571
90			1	CMI-30			*Pelobacter propionicus* strain DSM 2379 (CP000482)	83	*Proteobacteria (Delta)*	549
91			1	CMI-31			*Steroidobacter denitrificans* strain FS (EF605262)	93	*Proteobacteria (Gamma)*	646
92			1	CMI-32			*Novosphingobium nitrogenifigens* strain Y88 (DQ448852)	97	*Proteobacteria (Alpha)*	520

Supplementary Table S1. (*continued*)

Clone-phylotype No.[a]	Reed		Japanese loosestrife		Pond water		Authentic species (Accession No.)	Identity (%)	Phylum (Class)	Length (bp)
	Total no. of clone	Name of representative clone	Total no. of clone	Name of representative clone	Total no. of clone	Name of representative clone				
93			1	CMI-33			*Steroidobacter denitrificans* strain FS (EF605262)	93	*Proteobacteria (Gamma)*	519
94			1	CMI-34			*Planctomyces limnophilus* strain IFAM 1008 (X62911)	92	*Planctomycetes*	600
95			2	CMI-35			*Novosphingobium pentaromativorans* strain US6-1 (AF502400)	98	*Proteobacteria (Alpha)*	580
96			1	CMI-37			*Novosphingobium aromaticivorans* strain DSM 12444 (CP000248)	96	*Proteobacteria (Alpha)*	626
97			1	CMI-38			*Lewinella agarilytica* strain SST-19 (AM286229)	85	*Bacteroidetes*	649
98			1	CMI-39			*Methylophilus leisingeri* strain DSM 6813 (AB193725)	95	*Proteobacteria (Beta)*	652
99			1	CMI-40			*Methylophilus leisingeri* strain DSM 6813 (AB193725)	96	*Proteobacteria (Beta)*	653
100			1	CMI-41			*Sporichthya polymorpha* strain DSM 46113 (X72377)	87	*Actinobacteria*	656
101			1	CMI-42			*Pseudomonas fluorescens* strain ATCC13525 (DQ207731)	88	*Proteobacteria (Gamma)*	657
102			1	CMI-43			*Steroidobacter denitrificans* strain FS (EF605262)	88	*Proteobacteria (Gamma)*	660
103			1	CMI-44			*Methylocella silvestris* strain BL2 (AJ491847)	96	*Proteobacteria (Alpha)*	514
104			1	CMI-45			*Devosia subaequoris* strain HST3-14^{T} (AM293857)	92	*Proteobacteria (Alpha)*	610
105			1	CMI-47			*Streptomyces hiroshimensis* strain NBRC 14693 (AB249979; *Actinobacteria*)	84	*Acidobacteria*	608
106			1	CMI-48			*Ralstonia insidiosa* strain CCUG 46388 (AJ539233)	93	*Proteobacteria (Beta)*	522
107			1	CMI-49			*Owenweeksia hongkongensis* strain UST20020801 (AB125062)	86	*Bacteroidetes*	399

Supplementary Table S1. (*continued*)

Clone-phylotype No.[a]	Reed		Japanese loosestrife		Pond water		Authentic species (Accession No.)	Identity (%)	Phylum (Class)	Length (bp)
	Total no. of clone	Name of representative clone	Total no. of clone	Name of representative clone	Total no. of clone	Name of representative clone				
108			1	CMI-50			*Niastella jeongjuensis* strain GR20-13 (DQ244076)	93	*Bacteroidetes*	565
109			1	CMI-51			*Persephonella guaymasensis* strain H2 (AF385630; *Aquificales*)	78	*Planctomycetes*	647
110			1	CMI-53			*Desulfomonile limimaris* (AF230531; *Proteobacteria*)	79	*Acidobacteria*	532
111			1	CMI-54			*Polyangium vitellinum* strain Pl vt1 (AJ233944)	89	*Proteobacteria (Delta)*	630
112			2	CMI-55			*Planctomyces maris* strain DSM 8797^{T} (AJ231184)	85	*Planctomycetes*	653
113			1	CMI-56			*Devosia subaequoris* strain HST3-14^{T} (AM293857)	93	*Proteobacteria (Alpha)*	640
114			2	CMI-57			*Devosia subaequoris* strain HST3-14^{T} (AM293857)	93	*Proteobacteria (Alpha)*	599
115			2	CMI-59			*Hyphomicrobium zavarzinii* strain ZV-622 (Y14305)	93	*Proteobacteria (Alpha)*	598
116			1	CMI-60			*Hyphomicrobium vulgare* strain ATCC 27500 (Y14302)	94	*Proteobacteria (Alpha)*	547
117			1	CMI-61			*Methylocapsa acidiphila* strain B2^{T} (AJ278726)	97	*Proteobacteria (Alpha)*	650
118			1	CMI-62			*Haliscomenobacter hydrossis* strain DSM 1100 (AJ784892)	85	*Bacteroidetes*	542
119			1	CMI-63			*Bradyrhizobium elkanii* strain strain USDA 76 (U35000)	100	*Proteobacteria (Alpha)*	567
120			1	CMI-64			*Porphyrobacter neustonensis* strain DSM 9434^{T} (AF465838)	98	*Proteobacteria (Alpha)*	429
121			1	CMI-66			*Solirubrobacter soli* strain Gsoil 355 (AB245334)	88	*Actinobacteria*	548
122			1	CMI-67			*Sphingopyxis flavimaris* strain SW-151 (AY554010)	96	*Proteobacteria (Alpha)*	632
123			1	CMI-68			*Clostridium putrefaciens* strain DSM 1291 (AF127024; *Firmicutes*)	83	*Acidobacteria*	624

Supplementary Table S1. (*continued*)

Clone-phylotype No.[a]	Reed		Japanese loosestrife		Pond water		Authentic species (Accession No.)	Identity (%)	Phylum (Class)	Length (bp)
	Total no. of clone	Name of representative clone	Total no. of clone	Name of representative clone	Total no. of clone	Name of representative clone				
124			1	CMI-69			*Steroidobacter denitrificans* strain FS (EF605262)	94	*Proteobacteria (Gamma)*	540
125			1	CMI-70			*Phenylobacterium koreense* strain Slu-01 (AB166881)	98	*Proteobacteria (Alpha)*	648
126			1	CMI-71			*Rhodobacter sphaeroides* strain ATCC 17023^{T} (DQ342321)	97	*Proteobacteria (Alpha)*	576
127			1	CMI-72			*Hespellia porcina* strain PPC80 (AF445239; *Firmicutes*)	82	Candidate phylum OP10	572
128			1	CMI-73			*Desulfonatronum cooperativum* strain Z-7999 (AY725424; *Proteobacteria*)	82	*Acidobacteria*	592
129			1	CMI-74			*Planctomyces brasiliensis* strain DSM 5305^{T} (AJ231190)	85	*Planctomycetes*	558
130			1	CMI-75			*Flavobacterium ferrugineum* strain DSM 30193^{T} (AM230484)	94	*Bacteroidetes*	609
131			1	CMI-77			*Desulfovibrio intestinalis* strain KMS2 (Y12254; *Proteobacteria*)	78	Unclassified	524
132			1	CMI-78			*Spirulina laxissima* strain SAG B256.80 (Y18798; *Cyanobacteria*)	74	*Planctomycetes*	604
133			1	CMI-79			*Pirellula staleyi* strain ATCC 35122 (AF399914)	87	*Planctomycetes*	638
134			1	CMI-80			*Geopsychrobacter electrodiphilus* strain A2 (AY187304; *Proteobacteria*)	82	Unclassified	545
135			1	CMI-81			*Flavisolibacter ginsengisoli* strain Gsoil 643 (AB267477)	94	*Bacteroidetes*	631
136			1	CMI-82			*Hyphomicrobium zavarzinii* strain ZV-622 (Y14305)	88	*Proteobacteria (Alpha)*	666
137			1	CMI-83			*Nitrincola lacisaponensis* strain 4CA (AY567473)	86	*Proteobacteria (Gamma)*	649
138			1	CMI-85			*Sorangium cellulosum* strain DSM14627 (EU240497)	85	*Proteobacteria (Delta)*	797

Supplementary Table S1. (*continued*)

Clone-phylotype No.[a]	Reed		Japanese loosestrife		Pond water		Authentic species (Accession No.)	Identity (%)	Phylum (Class)	Length (bp)
	Total no. of clone	Name of representative clone	Total no. of clone	Name of representative clone	Total no. of clone	Name of representative clone				
139					1	CKW-1	*Polynucleobacter necessarius* strain STIR1 (CP001010)	99	*Proteobacteria (Beta)*	462
140					1	CKW-2	*Aquaspirillum delicatum* strain LMG 4328 (AF078756)	96	*Proteobacteria (Beta)*	580
141					2	CKW-3	*Streptacidiphilus jiangxiensis* strain 22202 (AY389139)	91	*Actinobacteria*	580
142					1	CKW-4	*Leifsonia poae* strain DSM 15202 (AM410682)	95	*Actinobacteria*	438
143					1	CKW-5	*Leifsonia aquatica* strain JCM 1368 (D45057)	95	*Actinobacteria*	567
144					3	CKW-6	*Aquaspirillum delicatum* strain LMG 4328 (AF078756)	97	*Proteobacteria (Beta)*	534
145					1	CKW-7	*Aquaspirillum delicatum* strain LMG 4328 (AF078756)	96	*Proteobacteria (Beta)*	574
146					1	CKW-8	*Acidovorax facilis* strain LMG 2193 (EU024133)	96	*Proteobacteria (Beta)*	583
147					2	CKW-16	*Acidovorax facilis* strain LMG 2193 (EU024133)	96	*Proteobacteria (Beta)*	583
148					3	CKW-17	*Acidovorax facilis* strain LMG 2193 (EU024133)	98	*Proteobacteria (Beta)*	582
149					2	CKW-18	*Aquaspirillum delicatum* strain LMG 4328 (AF078756)	98	*Proteobacteria (Beta)*	550
150					6	CKW-19	*Tetrasphaera vanveenii* strain Ben 70 (DQ007320)	91	*Actinobacteria*	570
151					3	CKW-20	*Cetobacterium somerae* strain WAL 14325 (AJ438155)	99	*Fusobacteria*	593
152					6	CKW-29	*Arcocella aquatica* strain NO-502^{T} (AJ535729)	90	*Bacteroidetes*	501
153					3	CKW-30	*Emticicia oligotropha* strain GPTSA100-15 (AY904352)	97	*Bacteroidetes*	583
154					1	CKW-31	*Emticicia oligotropha* strain GPTSA100-15 (AY904352)	98	*Bacteroidetes*	602

Supplementary Table S1. (*continued*)

Clone-phylotype No.[a]	Reed		Japanese loosestrife		Pond water		Authentic species (Accession No.)	Identity (%)	Phylum (Class)	Length (bp)
	Total no. of clone	Name of representative clone	Total no. of clone	Name of representative clone	Total no. of clone	Name of representative clone				
155					1	CKW-35	*Aquaspirillum delicatum* strain LMG 4328 (AF078756)	98	*Proteobacteria (Beta)*	602
156					1	CKW-38	*Owenweeksia hongkongensis* strain UST20020801 (AB125062)	89	*Bacteroidetes*	590
157					1	CKW-46	*Flexibacter canadensis* strain IFO 15130 (AB078046)	86	*Bacteroidetes*	456
158					1	CKW-47	*Legionella taurinensis* strain ATCC 700508 (DQ667196)	93	*Proteobacteria (Gamma)*	480
159					1	CKW-59	*Emticicia oligotropha* strain GPTSA100-15 (AY904352)	97	*Bacteroidetes*	580
160					1	CKW-60	*Limnobacter thiooxidans* strain CS-K2 (AJ289885)	94	*Proteobacteria (Beta)*	521
161					1	CKW-62	*Methylophilus methylotrophus* strain NCIMB 10515 (AB193724)	95	*Proteobacteria (Beta)*	627
162					1	CKW-71	*Niastella jeongjuensis* strain GR20-13 (DQ244076)	93	*Bacteroidetes*	572
163					1	CKW-72	*Acidovorax konjaci* strain ICMP 7733 (AF137507)	98	*Proteobacteria (Beta)*	562
164					1	CKW-75	*Fluviicola taffensis* strain RW262 (AF493694)	92	*Bacteroidetes*	572
165					1	CKW-76	*Fluviicola taffensis* strain RW262 (AF493694)	93	*Bacteroidetes*	558
166					1	CKW-77	*Rhodoferax ferrireducens* strain T118[T] (CP000267)	98	*Proteobacteria (Beta)*	538
167					1	CKW-82	*Aquaspirillum delicatum* strain LMG 4328 (AF078756)	96	*Proteobacteria (Beta)*	522
168					1	CKW-83	*Cytophaga fermentans* strain ATCC 19072 (M58766)	87	*Bacteroidetes*	602
Total	85		85		85					
Novel microbes[b]	50 (40)		65 (57)		34 (16)					

[a] The clonal phylotypes whose sequences indicated less than 95% identity with those from authentic species are underlined.
[b] The number of phylotypes showing phylogenetic novelty was provided in parentheses.
